# Benefits of whole-body vibration to people with COPD: a community-based efficacy trial

**DOI:** 10.1186/1471-2466-14-38

**Published:** 2014-03-08

**Authors:** Trentham Furness, Corey Joseph, Geraldine Naughton, Liam Welsh, Christian Lorenzen

**Affiliations:** 1 School of Nursing, Midwifery & Paramedicine, Australian Catholic University, Fitzroy, Australia; 2 School of Exercise Science, Australian Catholic University, Fitzroy, Australia; 3 NorthWestern Mental Health, 1 North, City Campus, The Royal Melbourne Hospital, Grattan Street, Parkville, Victoria 3050, Australia; 4 Centre for Sports and Exercise Medicine, Queen Mary University of London, London, UK; 5 Department of Respiratory Medicine, Royal Children’s Hospital, Melbourne, Australia

**Keywords:** COPD, Exercise, Whole-body vibration, Functional performance, Lower limbs, Functional independence

## Abstract

**Background:**

Benefits of community-based whole-body vibration (WBV) as a mode of exercise training for people with chronic obstructive pulmonary disease (COPD) have not been investigated. The low skill demand of WBV may enhance habitual sustainability to physical activity by people with COPD, provided efficacy of WBV can be established. The purpose of this trial was to compare a community-based WBV intervention with a sham WBV (SWBV) intervention and monitor exacerbations, exercise tolerance, and functional performance of the lower limbs of people with COPD.

**Methods:**

Community-dwelling adults with a GOLD clinical diagnosis of COPD were recruited to the trial. This was a Phase II efficacy trial with crossover to sham intervention interspersed with two-week washout. Each six-week intervention consisted of two sessions per week of either WBV or SWBV. The interventions were completed in the home of each participant under supervision. The outcome measures were selected psychological (perceived dyspnoea) and physiological (heart rate and oxygen saturation) responses to exercise, simulated activities of daily living (timed-up-and got test and 5-chair stands test), and selected kinematic variables of gait across the 14-week trial.

**Results:**

Sixteen adults with stable COPD were recruited to the trial. No exacerbations were reported during the WBV or SWBV interventions. After WBV, performance of activities of daily living (ADLs) and gait improved (*p* ≤ 0.05), while there was no change after SWBV (*p* > 0.05). Despite five withdrawals during the washout period, a 100% compliance to each six-week intervention was noted.

**Conclusions:**

Results showed that WBV did not exacerbate symptoms of COPD that can be associated with physical inactivity. The WBV intervention improved tests to simulate ADLs such as rising from a chair, turning, and walking gait with greater effect than a SWBV intervention. If a placebo effect was systemic to the WBV intervention, the effect was negligible. As a standalone community-based intervention, WBV was an efficacious mode of exercise training for people with stable COPD that did not negatively effect exercise tolerance or exacerbate the disease, while concurrently improving functional performance of the lower limbs.

**Trial registration:**

Australian and New Zealand Clinical Trials Registry ACTRN12612000508875.

## Background

As a cornerstone of COPD management, a minimum goal of pulmonary rehabilitation is the maintenance of exercise tolerance and performance of activities of daily living (ADLs) [1]. As components of pulmonary rehabilitation, efficacy of modes of resistance training and aerobic conditioning have been described along with improvement in exercise tolerance [2]. However, the associated perceived dyspnoea and hypoxemia can limit compliance to pulmonary rehabilitation interventions [3] and as such, the need for safe and valid exercise interventions specifically for people with COPD are salient. For initiatives to assist the promotion of habitual and sustainable exercise training and reduce the burden of COPD, a strong evidence base supporting program design is needed.

Two common modes of exercise training; aerobic conditioning and resistance training, routinely exacerbate dyspnoea for people with COPD and may lead to reduced physical activity because of fear of breathlessness [4]. The clinical and social merit of modes of exercise training that can minimise dyspnoea may add to the benefit of pulmonary rehabilitation interventions for people with COPD.

Recently, effects of whole-body vibration (WBV) on muscular strength and muscular power of the lower limbs of people with COPD have been investigated [5,6]. Coupled with pulmonary rehabilitation, WBV may have enhanced exercise tolerance, and did not elicit exacerbations of the disease [5]. As a standalone out-patient intervention, WBV improved functional capacity of people with COPD [6]. However, efficacy of WBV as a standalone community-based mode of exercise training attractive to individuals after structured pulmonary rehabilitation and other modes of out-patient care requires further investigation.

It remains unknown if a long-term community-based WBV intervention would elicit exacerbations of COPD, such as increased dyspnoea or hypoxemia, or if a WBV intervention would be well tolerated and adhered to by community-dwelling adults with stable COPD. As such, the aim of this Phase II efficacy trial was to report long-term benefits of a standalone community-based WBV intervention on performance of ADLs and gait by people with COPD as well as acute effects on dyspnoea, heart rate, and oxygen saturation.

## Methods

### Design

This Phase II efficacy trial was competed with a non-randomised, cross-over design to sham [7]. Participants with COPD provided informed consent to be allocated first to; (1) a six-week WBV intervention, and then (2) a six-week sham WBV (SWBV) intervention. A two-week washout interval interspersed the WBV and SWBV interventions. Across the 14-week trial period, data were collected at baseline and subsequent fortnights. Participants completed two training sessions per week. Participants agreed not to commence physical activity beyond their usual routine across the 14-week duration of the trial. The trial was approved by the Southern Health Human Research Ethics Committee A and the Australian Catholic University Human Research Ethics Committee. The trial is registered with the Australian New Zealand Clinical Trials Registry (ACTRN12612000508875).

### Participants

Patients of the Department of Sleep and Respiratory Medicine, Monash Medical Centre, with a GOLD diagnosis of COPD were contacted via introductory letter to peruse interest in the trial. For inclusion to the trial, potential participants had to exhibit stable COPD, be living in a fully-independent residence, and have the capacity to complete ADLs [7,8]. Potential participants also had to be free of self-reported WBV contraindications [9] and successfully complete a battery of balance, vision and cognition tests [8]. Potential participants were excluded if they were currently treated with corticosteroids or had experienced self-reported COPD exacerbations earlier than six months prior to the commencement of the trial.

### Independent and dependent variables

The independent variables were ‘intervention’ with two levels: (1) WBV and, (2) SWBV, and ‘test occasion’ with three levels: (1) pre-test, (2) mid-test, and (3) post-test. The dependent variables were: (1) acute exercise tolerance (rating of perceived dyspnoea, heart rate, and oxygen saturation), and (2) long-term functional performance (timed-up-and-go test, 5-chair stands test, stride length, stride time, and stride velocity). Acute data were collected prior to and during the final WBV or SWBV bout [7,10]. Long-term data were collected at least 48 hours after a WBV or SWBV bout [8,11].

Acute exercise tolerance was quantified with the Borg CR-10 VAS assessment of perceived breathing limitation [12]. Heart rate, oxygen saturation, and blood pressure were also quantified, with the CARESCOPE™ V100 Vital Sign Monitor (GE Health Care, Milwaukee, USA). Reliability of the test procedures was no less than ‘acceptable’ (ICC ≥ 0.700) for the three dependent variables [10].

The timed-up-and-go test (TUG) and 5-chair stands test (5-chair) were used to simulate ADLs and describe long-term functional performance. Kinematic variables of gait: stride length, stride time, and stride velocity were quantified with a GAITRite**^®^** Electronic Walkway (CIR Systems Inc, Peekskill, USA). With the methods described in our study protocol [7], reliability of the test procedures was no less than ‘good’ (ICC ≥ 0.881) for the five dependent variables.

### Interventions

The same side alternating vibration platform (Amazing Super Health, Melbourne, AUS) was transported to the homes of the participants for each training session. For each WBV bout, the vibration platform frequency was set at 25 Hz with a peak-to-peak displacement at 2.0 mm, peak acceleration was ~24.67 m.s^-2^, and the resulting gravitational force was ~2.52 *g*. Validity of the vibration platform peak-to-peak displacement and frequency were established prior to commencement of the trial [13]. A prototype vibration platform was developed to deliver the SWBV intervention. For each SWBV bout, the vibration platform frequency was 25 Hz and peak-to-peak displacement was ~0.0 mm, peak acceleration was ~0.00 m.s^-2^, gravitational force was ~0.0 *g* (cognisant that the Earth’s gravitational force is constant 1.0 *g*). When the prototype vibration platform was operational, the participants could hear the motor running and may have felt vibration. Participants were told the SWBV intervention was an “ultra-low frequency” vibration intervention that is “very different” to the WBV intervention. The participant wore flat soled shoes. Skidding was checked according to the recommended method of the International Society of Musculoskeletal and Neuronal Interactions [14]. Participants were told to stand with a bent knee posture that could be maintained for the duration of each WBV and SWBV bout. Knee flexion was checked manually with a goniometer after 30 seconds of every bout. During WBV, the participants stood with the knees flexed to 53° (SD ± 10°). During SWBV, participants stood with the knees flexed to 44° (SD ± 14°).

### Sample size and data analysis

Sample size with power computations were described previously [7,8]. In brief, sample size needed (based on paired *t*-test with α = 0.05, β = 0.90, and *n* = 14) was *n* = 16 for the 5-chair test and *n* < 10 for the TUG test. A sample of 16 participants was required based on the simulated ADL dependent variables. After normality was confirmed [15,16] repeated measures analysis of variance with repeated contrasts were computed for acute and long-term data for both WBV and SWBV with the Statistical Package for Social Scientists (SPSS Version 19.0, Chicago IL™). Effect size was described as Partial eta-squared (Partial η^2^). For long-term functional performance results, significance was accepted at *p* ≤ 0.05. To minimize potential of familywise error rate of long-term gait results, significance was accepted at *p* ≤ 0.02.

## Results

**Figure 1:**
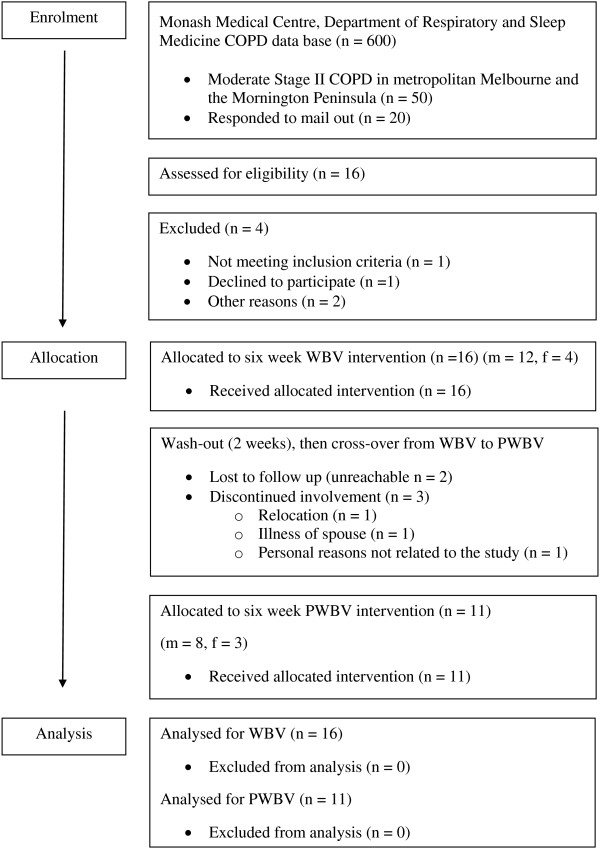
Flow of participation in the trial.

A total of 15 participants with GOLD Stage II COPD and one participant with GOLD Stage III COPD were recruited to the trial with 100% compliance to the both interventions (Figure 1). Demographic and disease characteristics are presented in Table 1. Acute and long-term results are shown in Table 2.

**Table 1:** Demographic and disease characteristics of the participants with COPD

**Descriptor**	**Mean**	**SD**
Age (years)	72	7
Stature (m)	1.71	0.1
Mass (kg)	85.7	20.4
BMI (kg.m-2)	29.3	6.1
SBP (mmHg)	139	13
DBP (mmHg)	75	10
Resting HR (beats.min-1)	82	10
FEV1 (L BTPS)	1.7	0.7
FEV1 % predicted	58.5	19.0
FVC (L BTPS)	3.0	0.8
FVC % predicted	83.2	15.0
FER %	52.3	10.8
PEF (L.sec-1)	4.2	0.8

N = 16 (4 females, 12 males). BMI: body mass index. SBP: systolic blood pressure. DBP: diastolic blood pressure. HR: heart rate. FEV1: forced expired volume of oxygen in the first second. FVC: forced vital capacity of the lungs. FER: forced expiratory ratio (FEV1.FVC-1). PEF: peak expiratory flow. L BTPS: Litres Body Temperature Pressure Saturated. Pack years range was 10.5 to 140 pack years.

**Table 2:** Acute and long-term results

**WBV**							**PWBV**					
	**Baseline**		**Week 3**		**Week 6**		**Week 9**		**Week 11**		**Week 14**	
Borg CR-10 VAS	1(1)	2(2)	2(1)	1(1)	1(1)	2(1)	2(1)	2(2)	2(1)	2(1)	2(1)	2(1)
HR (beats min^-1^)	82(10)	92(10)*	86(11)	94(12)*	83(11)	95(9)*	81(13)	88(13)	83(10)	88(13)	83(11)	90(8)
SpO_2_ (%)	97(2)	96(2)	96(2)	96(2)	95(2)	96(1)	95(2)	96(2)	96(2)	96(1)	94(2)	95(2)
TUG (sec)	11.3 (1.9)		10.7(1.7)*		9.8(1.9)*		10.8(2.2)		10.7(3.9)		10.6(1.7)	
5-chair (sec)	18.5(3.4)		16.4(2.7)*		15.1(2.4)*		16.1(2.4)		16.3(3.5)		16.3(2.8)	
SL (m)	1.14(0.16)		1.19(0.17)+		1.27(0.11)+		1.22(0.11)		1.20(0.13)		1.22(0.30)	
ST (sec)	1.11(0.13)		1.09(0.12)+		1.10(0.06)+		1.03(0.10)		1.02(0.09)		1.02(0.11)	
SVel (m.sec^-1^)	1.04(0.15)		1.10(0.15)+		1.16(0.11)+		1.19(0.15)		1.18(0.16)		1.20(0.16)	

Results are mean (SD). Pre: pre bout. During: during the bout. HR: heart rate. SpO2: oxygen saturation. TUG: timed-up-and-go test. %-chair: 5-chair stands test. SL: stride length. ST: stride time. SVel: stride velocity. *p ≤ 0.05. + *p* ≤ 0.02.

There was no acute effect of WBV or SWBV on rating of perceived dyspnea and oxygen saturation (*p* > 0.05) (Table 1). The WBV intervention increased heart rate by as much as 12 beats.min^-1^ (*p* ≤ 0.05). The SWBV intervention increased heart rate by as much as 7 beats.min^-1^ (*p* > 0.05).

Whole-body vibration improved performance of the TUG (13%) and 5-chair tests (18%) (*p* = 0.01). Across the first three weeks of WBV, TUG improvement was 0.5 sec (Partial η^2^ = 0.28, power = 0.99) and then an additional 0.9 sec (Partial η^2^ = 0.41, power = 0.95) after six weeks. Whole-body vibration also improved performance of the 5-chair test after three weeks by 2.1 sec (Partial η^2^ = 0.50, power = 0.95) and at Week 6 by 1.4 sec (Partial η^2^ = 0.49, power = 0.94). There was no effect of SWBV on simulated ADLs (*p* > 0.33). Performance was within ± 0.2 sec for both the TUG (2%) and 5-chair (1%) tests. Kinematic variables of gait improved after WBV (*p* = 0.01). Effect size was at least Partial η^2^ = 0.24 for stride length, Partial η^2^ = 0.14 for stride time, and Partial η^2^ = 0.72 for stride velocity. There was no effect of SWBV in kinematic variables of gait (*p* > 0.10).

## Discussion

The findings of this Phase II efficacy trial supported the use of a standalone community-based WBV intervention to improve functional performance of the lower limbs of people with COPD while avoiding exacerbations of the disease. Furthermore, WBV was more effective than SWBV for improvement of functional performance, yet comparable for acute markers of exercise tolerance. These findings support our earlier proof-of-concept trial of WBV as a mode of dyspnoea free physical activity [10].

### Exercise tolerance

Results of this trial compare favorably with other investigations of resistance training and aerobic conditioning of people with COPD. After 12-weeks of both training modes, Borg CR-10 VAS values were not different within and among resistance training and aerobic conditioning for people with COPD [17]. However, during performance of the six-minute walk test, perceived dyspnoea increased to a value of ‘6’ [17]. Concurrently, oxygen saturation reduced from 95 to 87% [17] and has reduced to similar values across other exercise investigations of people with COPD [18,19]. The effect of WBV in this trial was within the > 4% drop in oxygen saturation used to define hypoxemia [18].

For people with mild to moderate COPD, long-term physical activity can positively affect metabolic processes through improved oxygen saturation [20]. For participants of this trial however, there was an absence of meaningful change in dyspnoea and oxygen saturation across the WBV intervention. As such, if metabolic processes of people with COPD increased due to WBV, the change was not represented with the Borg CR-10 VAS and SpO_2_. Metabolic activity however, was shown to increase as demonstrated by increased heart rate in this trial. Across the trial, heart rate increased more during WBV than during SWBV. Others have reported similar increases in heart rate during WBV for sub-optimal health [21] and healthy older adults [22]. Given the known and broad physiological responses of the human body to physical activity, WBV may be viewed as an effective mode to increase metabolic activity of people with COPD.

### Functional performance of the lower limbs

Improvements in performance of the TUG test (13%) and 5-chair test (18%) after WBV for people with COPD in this trial were greater than the respective 6% and 12% improvements reported after a two-month WBV intervention of healthy older adults [23]. Given the community dwelling sub-optimal health status of participants in this trial, it is likely that the potential for improvement in performance of ADLs in people with COPD after WBV would be larger.

A three-week resistance training combined with WBV intervention for people with COPD reduced time taken to complete the 5-chair test by 4.0 sec [5]. Results of this efficacy trial show a 3.4 sec reduction of performance of the 5-chair test after a six-week standalone WBV intervention. For people with COPD, chair stands in 60 seconds and stair climbing have improved after resistance training and aerobic conditioning [24-26]. Compared with resistance training, WBV is not as effective for people with COPD because improvement of TUG test performance was 67% after 12 weeks of resistance training [27] compared with 13% in this trial. However, to achieve such large improvements, the resistance training intervention required supervision and equipment not usually available in the home.

### Gait

Previously, WBV with pulmonary rehabilitation [5] and as a standalone out-patient intervention [6] improved performance of the six-minute walk test. Participants in this trial walked with longer strides, in a faster time after the WBV intervention which may support previous findings. Similarly, eight weeks of combined resistance training and WBV in healthy older adults lengthened step length from 61 cm to 65 cm (7%) [28]. Stride length of participants in this trial was 13 cm longer (11%), which is similar to the ≥ 14 cm improvement of healthy older adults after resistance training interventions [29,30]. Similar effects were reported for gait velocity after resistance training and aerobic conditioning for people with COPD. Specifically, velocity increased 13%, from 0.89 m.sec^-1^ to 1.01 m.sec^-1^[25]. The 12% increase of stride velocity for people with COPD in this trial is similar to previous results in the range of 14 to 30% [24,27,31] for people with COPD. For healthy older adults, a 12% improvement in gait velocity after resistance training was described as an attractive strategy to improve gait [29]. When compared with this trial, WBV may also be an attractive strategy to improve gait for people with COPD.

Given the known physiological benefits of aerobic conditioning and resistance training on people with COPD (e.g., skeletal muscle hypertrophy and increased oxidative capacity), it may be appropriate to view possible mechanisms of improvement in functional performance of people with COPD after this trial with a similar perspective. However, given the predominance of Type II muscle fibre activity during exercise for people with COPD leading to increased anaerobic fatigue [32], it may be possible that improvement of functional performance of people with COPD after WBV could be partly attributed to training of Type II skeletal muscle fibres rather than for example, an increase of oxidative capacity at the muscle. Given the known leg fatigue exhibited by people with COPD due to high-intensity exercise designed to improve anaerobic performance, and persistence of poor exercise compliance associated with symptom limited participation [3,33], WBV may initially be a more attractive mode of exercise training for people with COPD.

### Compliance and drop-out

All participants successfully completed all WBV and SWBV sessions however, the number of participants reduced over the 14-week duration of this trial. The drop-out of participants was due to circumstances beyond the demands of the research during the washout period (Figure 1). As the importance of compliance to pulmonary rehabilitation had been documented and problematic, it was meaningful that participants complied 100% of the WBV intervention. As a mode of exercise training to maintain exercise tolerance, avoid exacerbations of COPD, and improve functional performance of the lower limbs, efficacy of this WBV intervention can be confirmed. Compared with other interventions, the 100% compliance in this trial was unique. The drop-out rate of three month resistance training research projects ranged from 20% to 38% [34,35]. Compliance, in some instances among healthy adults was at best 79% [36] and as low as 75% [34] for resistance training interventions. Symptom limitation was a major reason for poor compliance among people with COPD during pulmonary rehabilitation [3]. Given that only 48% of older Australians are physically active [37], the need of such activity that does not exacerbate people living with sub-optimal health is salient. The results of the community-based WBV intervention used in this trial confirm the ease and convenience of WBV highlighted by maximum compliance and an absence of drop-out during the WBV intervention. Compliance however, may have occurred due to the supervised nature of both the WBV and SWBV interventions. Because physical activity in the home is typically unsupervised, future randomised controlled trials of WBV and long-term unsupervised habitual sustainability should be conducted of people with COPD.

### Limitations

This trial was limited as participant; (1) nutrition, exercise history, motivation, and other environmental support mechanisms, and (2) current or past pharmacologic treatment for COPD (with the exception of corticosteroids) were not profiled. Furthermore, and similar with some resistance training interventions, the practical applicability of WBV as a standalone mode of exercise training in community-settings needs to be considered as the cost of a vibration platform may limit accessibility.

It may be that the participants reached a ‘ceiling’ that limited further improvement during SWBV and therefore, a placebo effect may have been systemic to the WBV intervention. It may also be that; (1) simply standing on the prototype vibration platform with the knees flexed was enough stimulus to maintain improvements after WBV, or (2) participants were more physically active after the WBV intervention despite the request not to begin any new mode of physical activity during the 14-week trial. Future research should focus about randomised controlled trials to more thoroughly describe effects of WBV and a potential placebo affecting people with COPD and describe the economic feasibility and long-term exacerbations of standalone community-based WBV interventions.

## Conclusions

This was the first trial to quantify and describe benefits of a standalone WBV intervention on exercise tolerance and functional performance of the lower limbs of people with COPD in a community-setting. Both the WBV and SWBV interventions were conducted in the home of each participant, which maximised participant compliance to each intervention. Results of this Phase II trial confirm efficacy of a WBV intervention to improve functional performance of the lower limbs of people with COPD. Whole-body vibration was a well-tolerated mode of exercise training for people with COPD highlighted by the absence of exacerbations of COPD.

## Competing interests

The authors declare that they have no competing interests.

## Authors’ contributions

All authors made substantial contribution to the conception and design of the trial, and preparation of this manuscript. TF recruited all participants, coordinated the trial, collected, and analysed all data. All authors give final approval of version to be published.

## Pre-publication history

The pre-publication history for this paper can be accessed here:

http://www.biomedcentral.com/1471-2466/14/38/prepub
